# When Cognitive Proximity Leads to Higher Evaluation Decision Quality: A Study of Public Funding Allocation

**DOI:** 10.3389/fpsyg.2021.697989

**Published:** 2021-10-22

**Authors:** Chuqing Zhang, Zheng Zhang, Daozhou Yang, Shayegheh Ashourizadeh, Lun Li

**Affiliations:** ^1^Economic Research Institute, Beijing Language and Culture University, Beijing, China; ^2^School of Journalism and Communication, Hubei University of Economics, Wuhan, China; ^3^Business Management and Organization, Wageningen University and Research, Wageningen, Netherlands; ^4^School of Economics and Management, Tsinghua University, Beijing, China

**Keywords:** cognitive proximity, decision-making quality, evaluation experience, evaluation effort, funding allocation

## Abstract

Project expert evaluation is the backbone of public funding allocation. A slight change in score can push a proposal below or above a funding line. Academic researchers have discovered many factors that may affect evaluation decision quality, yet the subject of cognitive proximity towards decision quality has not been considered thoroughly. Using 923 observations of the 2017 Beijing Innofund data, the study finds that cognitive proximity has an inverted “U-shape” relation to decision-making quality. Moreover, two contextual factors, evaluation experience and evaluation efforts, exert moderation effects on the inverted U shape. These findings fill the gaps in the current research on cognition-based perspective by specifying the mechanism of cognitive proximity in the evaluation field and contributing to improving decision-making quality by selecting appropriate evaluators. Theoretical contributions and policy implications have been discussed.

## Introduction

Project expert evaluation is the backbone of public funding allocation. In order to select the most innovative and promising project, grant funding agencies rely on project evaluation experts to decide which projects get funded. On such occasions, evaluators’ evaluation feedback and results are essential references for final resource allocation decisions (Olbrecht and Bornmann, 2010). Without high precision, some proposals will inevitably be incorrectly ranked and may undeservedly miss out on funding (Graves et al., 2011). Among the various factors that may affect decision quality, cognitive proximity towards decision quality has not been considered thoroughly, and the results remain inconsistent (Bornmann et al., 2010; Lee et al., 2013). Cognitive proximity refers to the degree of overlap between two actors concerning their knowledge bases (Wuyts et al., 2005; Broekel and Boschma, 2012). While it is supposed that cognitive proximity leads to higher decision quality (Dane et al., 2012; Li, 2017), other findings have reported opposite conclusions (Fisher and Keil, 2010; Mehta et al., 2011; Ottati et al., 2015). We boldly presume that neither too high nor too low proximity is suitable for decision quality. Too much proximity can be problematic because there is the risk for cognitive inertia, but too much distance is also problematic because of absorptive incapability (Nooteboom et al., 2007). Therefore, the degree to which an individual’s decision quality tends to rely on cognitive familiarity needs to be investigated, and how they make reviewers uncertain need to be further explored.

In the evaluation field, evaluators’ decision quality is also highly context-dependent. Factors like evaluation experience and efforts play indispensable roles during the evaluation process. Individuals with evaluation experience suggest that they can understand the underlying structural features of a problem, have superior pattern recognition skills, and develop more robust solutions to problems (North et al., 2009). On the other hand, the cognitive effort is the amount of attention devoted to creating a solution related to the intensity aspect of attention (Scheiter et al., 2020). It increases one’s cognitive-processing capacity to notice connections between different elements and make sense of these connections (Acar and Van den Ende, 2016). Nevertheless, there is no explicit evidence on the relationship between evaluation experience and efforts and decision quality. Most evidence is anecdotal, and there is surprisingly little compelling empirical evidence on this issue (Schuett, 2013). We believe that this issue is crucial because it addresses how the cognitive features of evaluators affect the quality of decisions. Under the influence of these two factors, individuals may become objectified, institutionalized, and embedded in their mental models and shape expectations and future interactions. Therefore, what kind of role the experience differences and evaluation efforts play in evaluation quality is critical.

This research addresses these issues by using a 923 sample size of Beijing Innofund. We find support for these arguments. Our results show cognitive familiarity has a curvilinear relation to decision-making quality, i.e., quality of decisions is highest at moderate levels of cognitive proximity, beyond which they recede. Moreover, the inverted U-shaped relationship between cognitive proximity and decision quality is moderated by evaluation experience and evaluation efforts.

This paper contributes several ways; we offer a more nuanced account of how the evaluator’s cognitive proximity affects decision quality. The results highlight that cognitive proximity has an inverted U-shape with decision quality, i.e., lesser expertise simply cannot see what experts can see, and highly close expertise may suffer knowledge boundedness (Boudreau et al., 2016). Moreover, our research contributes to a more comprehensive understanding of the impact of evaluation experience and evaluation efforts on the curvilinear relationship between cognitive proximity and quality of decisions by elucidating the moderating effects of evaluation experience and evaluation efforts.

## Theory and Hypotheses

### Expert Project Evaluation and Decision-Making Quality

Decision-making and decision-making competence are, at least to some extent, domain-dependent (Allwood and Salo, 2014). Decision-makers need to have a sufficient absorptive capacity to identify, interpret, and exploit knowledge of the target to make their predictions more reliable (Broekel and Boschma, 2012). Decision-makers, especially those close to a particular domain field, can observe and exploit a far broader array of informational cues. They perceive and appreciate more detail, complexity, patterns, and meaning when making the same observations as those not close to a certain field (Boudreau et al., 2016). These advantages in information processing are rooted in developing a richer, more textured library of domain-specific knowledge accumulated through extended periods of training, experience, and practice (Boudreau et al., 2016). When their mental model of the task is deficient initially, and subjects possess mental models that lack features needed to understand, control, and decide in problematic dynamic settings, decision-makers perform poorly.

Research on bounded rationality and expert cognition provides further explanation of decision making and its domain-dependent mechanism. Cognition is absorbing, interpreting, and categorizing knowledge (Kaplan and Tripsas, 2008). People bring a particular cognitive foundation along different life paths and environments to interpret, understand, and evaluate the world differently (Wuyts et al., 2005). These cognitive foundations form knowledge bases, which are the sources of expertise and action of individuals (Hautala, 2011), and lead to cognitive proximity between people (Nooteboom et al., 2007). Since evaluators have expertise in (or preferences for) one topic or approach (Li, 2017), they may not be able to identify fields of knowledge or research practices nor value the usefulness of the potential results of other areas (Banal-Estañol et al., 2019). Therefore, cognitive proximity is a key determinant towards decision-making quality.

Indeed, several studies have tried to discover the relationship between cognitive proximity and decision-making quality. For example, Dane (2010) supposes cognitive proximity may lead to entrenchment, which increases the difficulty of adaptation within one’s domain, causing evaluators to misjudge their option selection. Fisher and Keil (2010) discover that experts tend to overestimate their ability to explain their own areas more than they do unfamiliar areas. Mehta et al. (2011) demonstrate that experts’ higher sense of accountability for their judgments, coupled with their highly developed schemata, is identified as the mechanism of misjudgment. Mueller et al. (2012) demonstrate a negative bias against creativity when evaluators experience knowledge uncertainty. On the other hand, Li (2017) shows that evaluators are better informed but more biased about the quality of projects in their own area. Criscuolo et al. (2017) further discover that too large or too small a knowledge gap can cause decision-making bias. Therefore, the extent to which cognitive proximity may affect decision-making quality is needed to be explored. So, we aim to fill this gap by offering a more nuanced account of how evaluators’ decision quality relies on cognitive familiarity in an inverted U-shape.

### Cognitive Proximity and Decision Quality

Intellectual distance and uncertainty might reduce decision quality in assessments (Boudreau et al., 2016). Distance from one’s knowledge domain reinforces the evaluator’s inability to assess the merits of knowledge correctly and increases difficulties in searching, internalizing, and leveraging that knowledge (Acar and Van den Ende, 2016). Thus, it makes it hard for individuals to make reliable predictions of that knowledge, and we might expect a greater uncertainty (Boudreau et al., 2016). In contrast, when evaluators have similar (but not necessarily identical) frames of knowledge, they can better understand the underlying structural features of a problem (North et al., 2009). Decisions will be easier, more predictable, and better understood when they continue from an existing body of well-established knowledge (Koehler, 1991).

However, if evaluators are too familiar with a particular field, i.e., their cognitive proximity is close enough to the rated project, they may process information in a manner that reinforces their prior opinion or expectation (Ottati et al., 2015). Evaluators with cognitive proximity tend to consider more attributes and attempt to conduct a more detailed comparative assessment in their evaluations. This effort will, in turn, cause them to align the non-alignable differences by filling in with their well-developed schemata (Mehta et al., 2011). Their complex schemata increase the likelihood of falsely recalling an associative link from memory (Baird, 2003). Evaluators’ knowledge structures further contribute to these false recalls since their richly developed schemata enable them to recall a comparable attribute more easily, albeit inaccurately. It leads evaluators to adopt a relatively dogmatic, closed-minded orientation (Ottati et al., 2015) and is predisposed to experience psychological insecurity.

The optimal level of cognitive proximity follows from the need to keep some cognitive distance (to stimulate new ideas through recombination) and to secure some cognitive proximity (to enable effective communication and knowledge transfer; Broekel and Boschma, 2012). A certain degree of dissimilarity in terms of know-how, know-what, and way of thinking can be fruitful for both parts of project evaluation. Evaluators deal with this problem by ensuring that they share a common knowledge database and allowing a certain degree of differences in the other dimensions of cognitive proximity (Huber, 2012). Therefore, we can surmise that the evaluator’s decision quality may peak at the middle-level of cognitive proximity. In other words, we presume,

*H1*: Evaluators’ cognitive proximity has a curvilinear relationship (inverted U-shape) with decision quality, such that the positive relationship between cognitive proximity and decision quality is attenuated when familiarity exceeds a certain high level.

### Interaction Effect of Cognitive Proximity and Experience on Decision Quality

Experience is an essential factor influencing decision-making (Mishra et al., 2015). Individuals with evaluation experience can render evaluators with a better understanding of evaluation indicators and applicants’ development and eliminate their inconsistent expectations of those applicants (Mishra et al., 2015). As a result, individuals with evaluation experience may have greater cognitive flexibility or the ability to recognize and integrate information (Furr et al., 2012).

High cognitive proximity, on average, means a better understanding of a problem and high efficiency of decision making. Therefore, for a high cognitive proximity case, evaluators with high evaluation experience are likely to have a higher decision quality than evaluators with average experience levels (Kotha et al., 2013). The low cognitive proximity case will likely involve very little common ground between evaluators and the target project (Cronin and Weingart, 2007). Evaluators may have difficulties in understanding the nuances of the target project with minimal overlap (Huber and Lewis, 2010). As argued above, evaluators with a high evaluation experience are likely to have developed requisite common ground among all projects, reducing uncertainties. Therefore, at low cognitive proximity, increasing evaluation experience is likely to impact the decision quality positively.

Conversely, decreasing evaluation experience will likely exacerbate cognitive dissonance with lower decision-making quality (Kotha et al., 2013). For the inverted U shape, this implies that when evaluation experience is high, evaluators are allowed to correctly identify the objectively maximizing option (Scheiter et al., 2020). This means that in our context, the curve flattens when evaluators’ evaluation experience is high. Schroter et al. (2004) show that a short training program to improve peer review was slightly effective. Schroter et al. (2008) conducted a randomized trial, and it showed that the performance of reviewers was improved with different types of training intervention. Therefore, we propose the following hypothesis,

*H2*: The inverted U-shaped relationship between cognitive proximity and decision quality is moderated by evaluation experience, such that the curvilinear relationship is less pronounced for evaluators with high evaluation experience than for those with low evaluation experience.

### Interaction Effect of Cognitive Proximity and Efforts on Decision Quality

Cognitive effort is inherent to task complexity and individual’s knowledge working on it (Scheiter et al., 2020). With more and more information gathered, the chance of forming a valid representation of a decision strongly increases (Blaywais and Rosenboim, 2019). As a result, evaluators might make optimal decisions. On the other hand, if evaluators are given insufficient examination time to consider which pieces of information are useful, one might expect them to conduct limited reviews of applications (Frakes and Wasserman, 2017). Thus, they may overlook relevant information and grant funding to unqualified proposals (Kim and Oh, 2017).

As stated above, high cognitive proximity means better understanding of a problem and high efficiency of decision making. In such conditions, high cognitive effort engagement may further improve the odds of making a reliable decision. For the low cognitive proximity case, evaluation effort is vital because it increases one’s cognitive-processing capacity to notice connections between different elements and to make sense of these connections in such a way that they can be recombined to generate a novel solution to a given problem (Li et al., 2013). If evaluators perceive their current state of knowledge as insufficient, they are proposed to exert cognitive effort to close the gap between actual and desired levels of decision quality. The inverted U shape implies that the curve becomes less pronounced when evaluators’ cognitive effort is high. Therefore, we propose the following hypothesis,

*H3*: The inverted U-shaped relationship between cognitive proximity and decision quality is moderated by evaluation efforts, such that the curvilinear relationship is less pronounced for evaluators with high evaluation efforts than for those with low evaluation efforts.

## Methodology

### Beijing Innofund Institution

A small and medium-sized technology-based enterprise special fund, *Beijing Innofund*, was initiated by the Government of Beijing Municipality in 2006. It aims to support small and medium-sized enterprises’ technological innovation activities and foster their growth in Beijing. Over 4,000 innovative SMEs have successfully won the grant, and 2,200 of them have grown into national high-tech enterprises.

In each year, there are thousands of firms applying for Beijing Innofund. Firms submit their proposals to one of the 10 panels: electronic information, biomedicine, new materials, equipment manufacturing, modern agriculture, sustainable development, clean energy and energy-saving, electric vehicles, cultural innovation, and city management. After qualification scrutiny, the evaluation process begins. Evaluators log into the evaluation system for online review. To ensure the fairness of evaluation, proposals are randomly distributed to evaluators. Evaluators make their own judgments independently, and no discussion group will be set up.

In particular, each proposal is rated by two technical evaluators and three business evaluators. Technical evaluators are generally researchers or CTOs who have deep insight into technology. Business evaluators are mainly composed of investors, entrepreneurs, or managers, who have made outstanding achievements in the business field. The evaluation indicators of technical evaluators are slightly different from business evaluators. Technical evaluators usually focus on the project’s technological innovation capabilities; their review indicators are human resources, technology innovation, and business model. Business evaluators focus on human resources, economic performance, product innovation, and business models. When the evaluation is completed, the system will automatically summarize all five evaluators’ scoring for the proposal. Their average score will be the final score, and it determines which proposals get the grant. Typically, around the top 23% of proposals in each panel will be the winners.

In 2017, nearly 2000 companies applied for Beijing Innofund, and over 200 evaluators participated in the evaluation process. In the end, 260 projects received support.

### Data and Sample

The data used in this paper are obtained from *Beijing Innofund* database. The database contains rich information about proposals, evaluators, and evaluation results. Although evaluators may belong to different affiliations, we prefer evaluators employed in academic departments (universities and research institutions). Part of the reason is that information of these types of evaluators is more conveniently supplemented, and part of the reason lies in that we are more curious about how knowledge proximity affects decision quality in these knowledge-intense departments. Therefore, by deleting unmatched and missing data, our research was based on a sample size of 923 experts–proposal scoring pairs consisting of 35 evaluators and 772 proposals in 2017.

### Measurements

#### Dependent Variable

##### Decision Quality

The higher the decision-making quality, the more reliable the decisions are. A high-quality decision should remain satisfying after the decision-maker decides, that is, s/he believes it is the right one (Allwood and Salo, 2014). Bruine de Bruin et al. (2007) developed a self-report measure of adverse decision outcomes. Milkman et al. (2009) suggested that decision quality can be evaluated based upon whether, after the fact, the decision-maker remains satisfied with his or her decisions. The approach taken in this study is based on a long tradition of research using subjective ratings for establishing predictive validity (Wood and Highhouse, 2014). Accordingly, we measured decision quality by asking the decision-maker whether s/he has confidence in their rating. They need to report it on the 6-point scale (1=*entirely unsure*, 2=*unsure*, 3=*slightly less unsure*, 4=*slightly sure*, 5=*sure*, 6=*entirely sure*).

#### Independent Variables

##### Cognitive Proximity

To measure cognitive proximity between the evaluator’s expertise and proposals, we should first specify evaluators’ knowledge expertise. Based on Jeppesen and Lakhani’s (2010) study, we categorized evaluators’ knowledge expertise into scales 1 to 3 (1=*outside my field of expertise*, 2=*at the boundary of my field of expertise*, 3=*inside my field of expertise*). The Beijing Innofund evaluator database contains four self-reported fields with which evaluators suppose they are most familiar, and we recoded those fields as three if they are coincident with projects’ panel field. We recorded those projects’ fields that matched reviewers’ majors but did not belong to the four most familiar fields as 2. Those fields that were not related to evaluator’s knowledge base were recoded as 1. The higher the value, the higher the cognitive proximity between the evaluator and the applicants.

##### Evaluation Experience

Evaluators’ evaluation experience was measured by the number of times evaluators had participated in Beijing Innofund evaluations. Due to Beijing Innofund having undergone a complete reform in 2015, its database only keeps evaluation information since 2015. Therefore, the maximum evaluation experience is 3 and the minimum is 1. A higher number means evaluators are more experienced.

##### Evaluation Efforts

Evaluation effort is a commonly used indicator of the amount of cognitive resources expended in a task. According to Acar and Van den Ende (2016), we took the total minutes evaluators had spent generating their final solution, including thinking about the solution and reading and researching it.

#### Control Variables

##### Gender

To provide equitable assessment, systematic differences in decisions by male and female evaluators need to be addressed (Tamblyn et al., 2018). We coded the male evaluator as 0; female evaluator as 1.

##### Education Level

Evaluators with higher degree level show more risk (Li, 2017), so there is a need to control it into the model. We coded bachelor as 1, master as 2, Ph.D. as 3.

##### Major

Discipline differences were apparent in evaluation studies (Bornmann et al., 2010); we divided the evaluator’s major into three categories, science and technology, liberal arts, and interdisciplinary backgrounds. Science and technology includes physics, chemistry, biology, engineering, astronomy, and mathematics; liberal arts includes literature, history, philosophy and art, human geography, law, education, economics, and management. If evaluators only major in science and technology, we coded it as 1; if evaluators only major in liberal arts, we coded it as 2; if a certain evaluator has both knowledge in science and technology and liberal arts, then we coded it as 3.

##### Evaluator’s Types

Since evaluation indicators of different types are different, evaluator types also need to be controlled (Jayasinghe et al., 2003). We coded the technical evaluator as 1; the business evaluator as 2.

##### Application Field

Applications and evaluators tend to be systematically different in each field (Banal-Estañol et al., 2019). We generate dummy variables for each field.

### Statistical Analysis

Since our dependent variable is a nonnegative count variable, the negative binomial model is appropriate for estimating it. The negative binomial model allows the variance to differ from the mean, which can correct for overdispersion. Moreover, since the evaluations of the same evaluator may not be independent, there is a need to control for evaluators’ fix effect. We use hierarchical models, with Model 1 serving as the baseline model that includes only the control variables, Models 2 to 3 introducing the independent variables, and Models 4 to 5 incorporating the moderating variables. Moreover, no symptoms of multicollinearity were observed, as the maximum variance inflation factor index does not exceed the critical value of 10.

## Results

Table 1 provides the means, standard deviations, and correlation coefficients of all study variables. It shows relatively moderate correlations among variables.

**Table 1:** Means, standard deviations, and correlation coefficients of all study variables.

		Mean	SD	Min	Max	1	2	3	4	5	6
1	Quality	4.982	0.887	1	6	1					
2	Familiarity	2.119	0.703	1	3	0.152^***^	1				
3	Experience	0.763	0.788	0	2	0.244^***^	0.246^***^	1			
4	Effort	1.414	1.191	0	4	0.134^***^	−0.204^***^	−0.006	1		
5	Gender	0.762	0.426	0	1	0.208^***^	0.301^***^	0.207^***^	0.091^***^	1	
6	Education	2.556	0.698	1	3	0.280^***^	0.130^***^	−0.109^***^	−0.046	−0.038	1

Standard errors in parentheses.

*** *p*<0.01.

### Main Effect

The results are given in Table 2. In Model 1, we enter the control variables (gender, education, major, types, and field). In Model 2 and Model 3, we include independent variables and their quadratic term to predict the curvilinear relationship that the evaluators’ cognitive proximity would have on assessing quality, respectively. The result of Model 2 shows a positive and non-significant effect of evaluation quality (*β*=0.018), and that the coefficient of the quadratic term of cognitive proximity is significant and negative (*β*=−0.082, *p*<0.1) in Model 3.

**Table 2:** Negative binomial regression models.

	Model 1	Model 2	Model 3	Model 4	Model 5
Gender	0.111^*^	0.107^**^	0.104^**^	0.083^**^	0.101^**^
	(0.057)	(0.051)	(0.042)	(0.033)	(0.041)
Education	0.085^**^	0.082^***^	0.070^***^	0.048^***^	0.070^***^
	(0.035)	(0.031)	(0.025)	(0.016)	(0.024)
Business evaluator	−0.284^***^	−0.293^***^	−0.329^***^	−0.337^***^	−0.327^***^
	(0.032)	(0.037)	(0.051)	(0.079)	(0.049)
Arts	−0.529^***^	−0.554^***^	−0.664^***^	−0.490^***^	−0.663^***^
	(0.055)	(0.072)	(0.110)	(0.113)	(0.111)
Interdiscipline	−0.588^***^	−0.603^***^	−0.674^***^	−0.473^***^	−0.677^***^
	(0.040)	(0.041)	(0.061)	(0.090)	(0.064)
Field	Controlled	Controlled	Controlled	Controlled	Controlled
Cognitive proximity		0.018	0.376^*^	0.620^***^	0.514^*^
		(0.023)	(0.209)	(0.212)	(0.279)
Cognitive proximity^2^			−0.082^*^	−0.128^***^	−0.115^**^
			(0.043)	(0.045)	(0.058)
Evaluation experience				0.720^***^	
				(0.273)	
Cognitive proximity^#^ Evaluation experience				−0.575^**^	
				(0.230)	
Cognitive proximity^2, #^ Evaluation experience				0.113^**^	
				(0.048)	
Effort					0.011
					(0.008)
Cognitive proximity^#^ effort					−0.012
					(0.007)
Cognitive proximity^2, #^effort					0.003^*^
					(0.002)
Constant	1.756^***^	1.757^***^	1.552^***^	1.146^***^	1.424^***^
	(0.157)	(0.147)	(0.192)	(0.276)	(0.262)

Standard errors in parentheses.

* *p*<0.1;

** *p*<0.05;

*** *p*<0.01.

**Figure 1:**
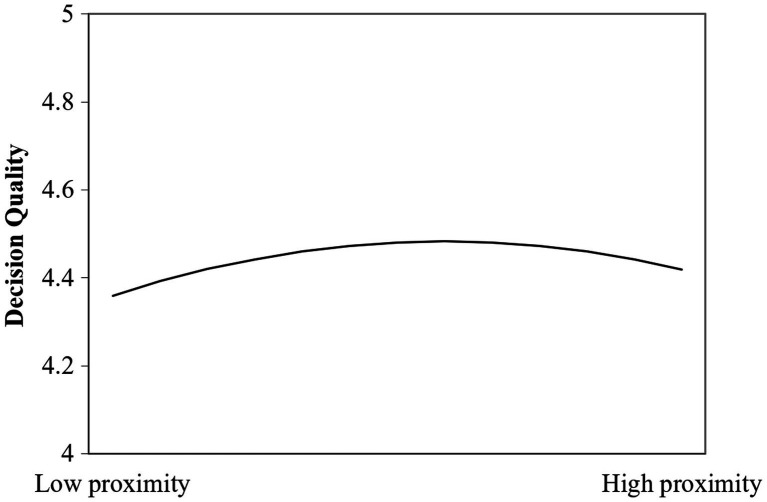
Cognitive proximity and decision quality.

To facilitate interpretation of the results, we plot in Figure 1 the relation of cognitive proximity and decision quality. The results indicate that once the evaluators’ knowledge familiarity reaches a certain level, decision quality peaks and declines as evaluators’ knowledge increases further. The inverted U shape of this curve is consistent with H1.

H2 predicts that the inverted U-shaped relationship between cognitive proximity and decision quality is moderated by evaluation experience, such that the curvilinear relationship is less pronounced for evaluators with high evaluation experience than those with low evaluation experience. We enter evaluation experience into the model in Model 4. The results of Model 4 reveal that the interaction term between cognitive proximity and assessing experience is negative and significant (*β*=−0.575, *p*<0.05), whereas the interaction term between the squared term of cognitive proximity and the assessing experience is positive and significant (*β*=0.113, *p*<0.05).

**Figure 2:**
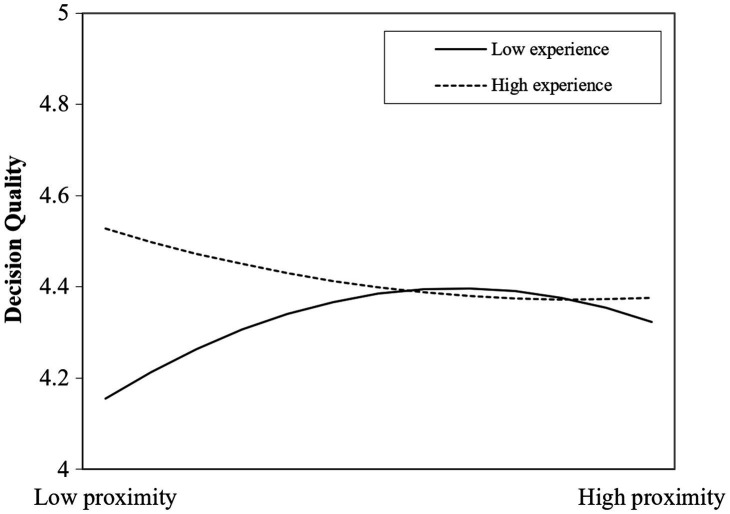
Moderating effects of evaluation experience on cognitive proximity and decision quality.

To facilitate interpretation, in Figure 2, we plot the curves between cognitive proximity and decision quality at a higher (one standard deviation above the mean) and a lower (one standard deviation below the mean) level of evaluation experience, respectively. The analysis suggests that for low and high cognitive proximity, the impact on assessing quality significantly differs between low and high assessing experience. For evaluators with low evaluation experience, cognitive proximity has an estimated increasingly positive effect on assessing quality. For high evaluation experience, cognitive proximity gradually drops on assessing quality, i.e., the increasing benefits of cognitive proximity on decision quality are lessened. However, after the turning point, the diminishing benefits of assessing quality are lessened for high experience evaluators since the curve turns up. Thus, the results prove that the inverted U-shaped relationship between cognitive proximity and decision quality is attenuated when the evaluator’s experience is high and accentuated when the experience is low.

H3 predicts that the inverted U-shaped relationship between cognitive proximity and decision quality is moderated by evaluation efforts, such that the curvilinear relationship is less pronounced for evaluators with high evaluation efforts than for those with low evaluation efforts. In Model 5, we include evaluation efforts and its interaction effect on decision quality with the squared term of familiarity. The interaction term between cognitive proximity and assessing efforts is negative but not significant with *β*=−0.012, whereas the interaction term between the squared term of cognitive proximity and the assessing effort is positive and significant (*β*=0.003, *p*<0.1).

**Figure 3:**
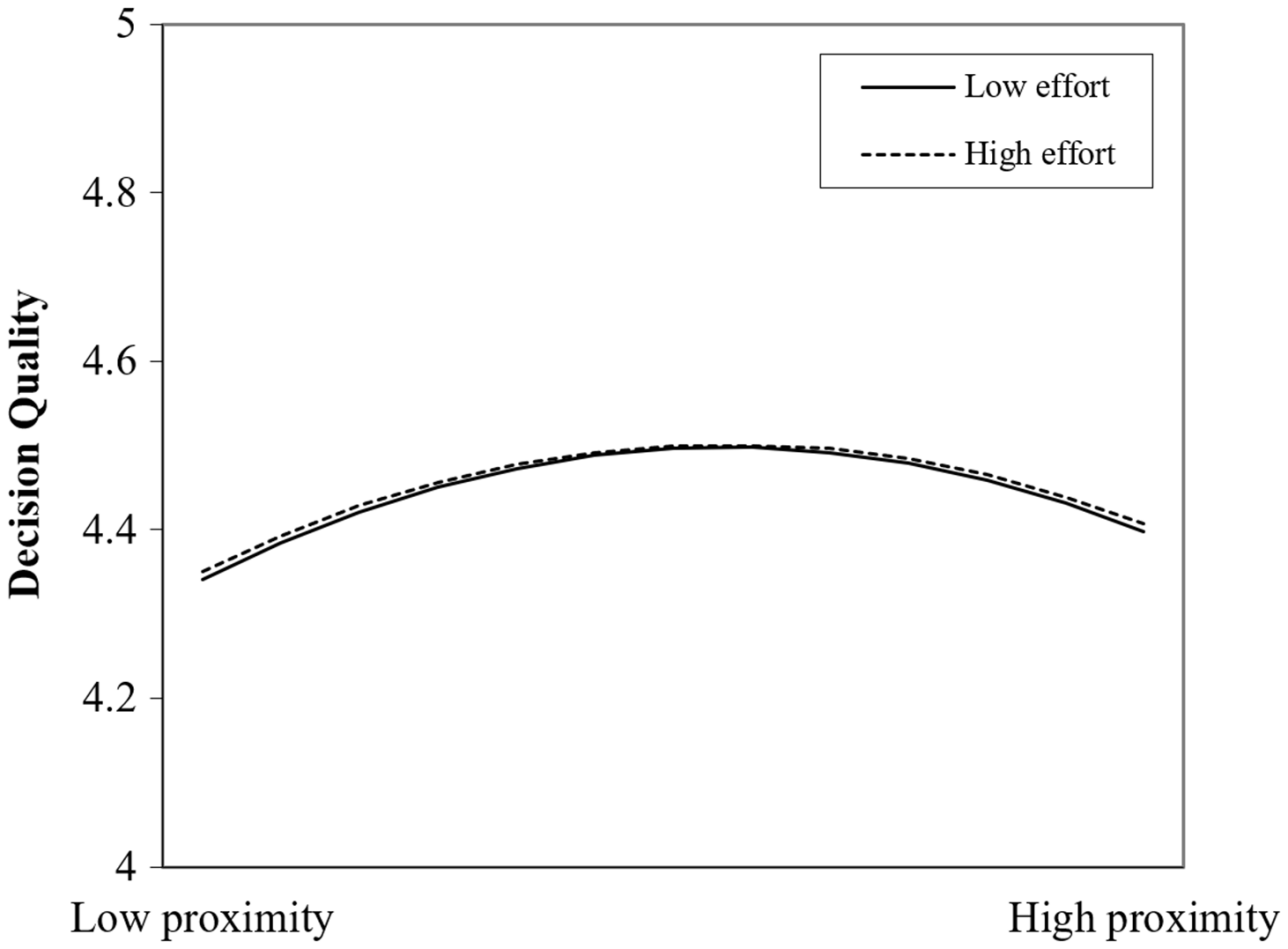
Moderating effects of evaluation efforts on cognitive proximity and decision quality.

Figure 3 plots the curves between cognitive proximity and decision quality at a higher (one standard deviation above the mean) and a lower (one standard deviation below the mean) level of evaluation efforts. The figure suggests that the impact of cognitive proximity on assessing quality slightly differs between low and high assessing efforts. The curvilinear relationship is less pronounced for evaluators with high evaluation efforts than those with low evaluation.

### Robust Check

We conduct further tests to check that our results are robust to changes in specifications (1) We conduct additional analysis by dropping the funded projects from the sample. Selecting unfunded projects can effectively reduce the error caused by quality issues between projects. Unfunded projects tend to be of similar quality, and their sample size is big enough (2) We replicate our findings with Poisson regression. This procedure is estimated using the maximum likelihood method compared to our main specification, effectively suppressing heteroscedasticity. Results provided in Table 3 are consistent with preliminary results.

**Table 3:** Poisson regression models.

	Unsupported				Supported			
	Model 1	Model 2	Model 3	Model 4	Model 5	Model 6	Model 7	Model 8
Gender	0.125^*^	0.121^**^	0.101^**^	0.117^**^	0.049^*^	0.042^**^	0.017	0.034^*^
	(0.068)	(0.053)	(0.042)	(0.051)	(0.026)	(0.021)	(0.018)	(0.020)
Education	0.092^**^	0.075^***^	0.051^***^	0.075^***^	0.062^***^	0.056^***^	0.039^***^	0.056^***^
	(0.041)	(0.029)	(0.018)	(0.028)	(0.021)	(0.019)	(0.014)	(0.019)
Business evaluator	−0.292^***^	−0.339^***^	−0.344^***^	−0.336^***^	0.052	0.001	−0.048	0.015
	(0.033)	(0.057)	(0.088)	(0.055)	(0.041)	(0.065)	(0.037)	(0.068)
Arts	−0.535^***^	−0.681^***^	−0.494^***^	−0.681^***^	−0.169^***^	−0.263^**^	−0.154^**^	−0.250^**^
	(0.057)	(0.123)	(0.122)	(0.124)	(0.062)	(0.110)	(0.077)	(0.124)
Interdiscipline	−0.620^***^	−0.714^***^	−0.502^***^	−0.719^***^	−0.119^***^	−0.168^***^	−0.015	−0.163^**^
	(0.046)	(0.070)	(0.103)	(0.071)	(0.040)	(0.061)	(0.058)	(0.070)
Field	Controlled	Controlled	Controlled	Controlled	Controlled	Controlled	Controlled	Controlled
Cognitive proximity		0.412^*^	0.673^**^	0.550^*^		0.230^*^	0.432^***^	0.452^*^
		(0.241)	(0.276)	(0.311)		(0.135)	(0.115)	(0.252)
Cognitive proximity^2^		−0.089^*^	−0.138^**^	−0.122^*^		−0.053^*^	−0.096^***^	−0.108^**^
		(0.050)	(0.058)	(0.065)		(0.029)	(0.030)	(0.053)
Evaluation experience			0.765^**^				0.585^***^	
			(0.342)				(0.144)	
Cognitive proximity^#^ Evaluation experience			−0.606^**^				−0.483^***^	
			(0.288)				(0.133)	
Cognitive proximity^2, #^ Evaluation experience			0.118^**^				0.100^***^	
			(0.059)				(0.030)	
Effort				0.011				0.017
				(0.008)				(0.014)
Cognitive proximity^#^ effort				−0.012				−0.019
				(0.007)				(0.012)
Cognitive proximity^2, #^effort				0.003^*^				0.005^*^
				(0.002)				(0.003)
Constant	1.725^***^	1.495^***^	1.054^***^	1.365^***^	1.536^***^	1.432^***^	1.149^***^	1.226^***^
	(0.191)	(0.243)	(0.367)	(0.311)	(0.096)	(0.095)	(0.121)	(0.177)
*N*	779	779	779	779	144	144	144	144

Standard errors in parentheses.

* *p*<0.1;

** *p*<0.05;

*** *p*<0.01.

Since proposal characteristics may also influence evaluation decisions, we opt for a robust cluster variance estimator to account for possible proposal correlations. Table 4 (within proposal) shows that the patterns of results were all consistent.

**Table 4:** Negative Binomial Regression within Proposals.

	Model 1	Model 2	Model 3	Model 4	Model 5
Gender	0.111^***^	0.107^***^	0.104^***^	0.083^***^	0.101^***^
	(0.018)	(0.017)	(0.016)	(0.014)	(0.016)
Education	0.085^***^	0.082^***^	0.070^***^	0.048^***^	0.070^***^
	(0.010)	(0.009)	(0.008)	(0.006)	(0.008)
Business evaluator	−0.284^***^	−0.293^***^	−0.329^***^	−0.337^***^	−0.327^***^
	(0.029)	(0.029)	(0.029)	(0.032)	(0.029)
Arts	−0.529^***^	−0.554^***^	−0.664^***^	−0.490^***^	−0.663^***^
	(0.038)	(0.039)	(0.046)	(0.048)	(0.047)
Interdiscipline	−0.588^***^	−0.603^***^	−0.674^***^	−0.473^***^	−0.677^***^
	(0.067)	(0.066)	(0.064)	(0.074)	(0.064)
Field	Controlled	Controlled	Controlled	Controlled	Controlled
Cognitive proximity		0.018^**^	0.376^***^	0.620^***^	0.514^***^
		(0.009)	(0.071)	(0.111)	(0.099)
Cognitive proximity^2^			−0.082^***^	−0.128^***^	−0.115^***^
			(0.015)	(0.024)	(0.021)
Evaluation experience				0.720^***^	
				(0.136)	
Cognitive proximity^#^ Evaluation experience				−0.575^***^	
				(0.116)	
Cognitive proximity^2, #^ Evaluation experience				0.113^***^	
				(0.024)	
Effort					0.011^**^
					(0.005)
Cognitive proximity^#^ effort					−0.012^**^
					(0.005)
Cognitive proximity^2, #^effort					0.003^***^
					(0.001)
Constant	1.756^***^	1.757^***^	1.552^***^	1.146^***^	1.424^***^
	(0.055)	(0.055)	(0.082)	(0.146)	(0.106)

Standard errors in parentheses.

^**^p<0.05;

*** *p*<0.01.

## Discussion

In this study, we aim to investigate the cognitive proximity effect in the decision-making field as well as their potential influential factors. We attempted to examine the extent to which one’s cognitive proximity would affect decision quality by testing the inverted U-shape relationship. Our hypothesis was supported. As explained by the cognitive-based perspective, decision-makers are more confident of their decision with intermediate levels of cognitive proximity.

However, according to Figure 1, the inverted U-shaped relation of cognitive proximity to decision quality is not apparent. When knowledge proximity increases from moderate to familiarity, evaluators’ quality of decision still maintains relatively high. We presume it lies in that when evaluators’ expertise increases, their psychological security may increase as well. In such conditions, evaluators do not need to make risky judgments, which are less easily threatened by uncertainty. It may induce them to overestimate the accuracy of their beliefs (Ottati et al., 2015). Therefore, the decreasing effect between cognitive proximity to decision quality may be lessened.

Moreover, the two moderators, evaluation experience and effort, both positively moderate decision quality. However, their efficacy is quite different. Evaluators’ performance in many fields relies on extensive practice and experience. It is proposed that information that is most confidently retrieved from memory, regardless of its accuracy, will be most influential in decision making (Cowley, 2004). The experience-based memory may help integrate the disparate elements of tasks that are not easily decomposed, permitting one to have a holistic judgment (Dane et al., 2012). Therefore, the effectiveness of experience is amplified at a high level of domain expertise (Dane et al., 2012). On the other hand, high cognitive proximity means better understanding of a problem and high efficiency of decision making. It is possible that many of the evaluators already have had an evident opinion on the topic, reflected in very high decision quality. In such conditions, evaluators may process information less effortfully than processing unfamiliar information (Acar and Van den Ende, 2016), so the curve is turning flatter. Thus, the effect of evaluation efforts on the inverted U-shaped relation is less significant than that of evaluation experience.

### Theoretical Implications

We challenge the traditional linear conceptualization of the effects of cognitive proximity on decision making and contribute to the literature on cognitive theory, peer evaluation, and decision theory by specifying the mechanism of cognitive proximity in the evaluation domain. Individuals with deep domain knowledge may have greater confidence in their predictions in fields where they have self-declared expertise (Heath and Tversky, 1991). Actors need to have a sufficient absorptive capacity to identify, interpret, and exploit knowledge (Cohen and Levinthal, 1990). However, too much cognitive proximity may result in cognitive lock-in, in the sense that too similar cognitive bases between evaluators and proposals may limit their cognition in verse. This study provides a cognitive account that may explain conflicting evidence about the link between cognitive proximity and decision quality by revealing that medium cognitive proximity is beneficial (Dane, 2010).

We also contribute to the decision-making field by exploring the consequences for evaluators to be exposed to experiences and effort. Evaluation experience has not received much research focus in the peer review literature; we highlight its importance in the evaluation field by elucidating its moderating role on the curvilinear relationship between cognitive proximity and decision certainty. We also identify that decision performance can be improved by allocating more cognitive resources to their execution by integrating evaluation efforts into cognition processes. Our research provides a new lens for investigating the impact of evaluation efforts on cognition and decision quality.

### Practical Implications

The funding agency should consider the cognitive proximity between the evaluators and the proposals to improve decision consistency and the quality of review decisions. It is also possible to supplement evaluations with statistics providing objective measures of the degree of familiarity for a given proposal (Boudreau et al., 2016). Another option for improvement involves balancing the characteristics of evaluators. For example, if an evaluator’s knowledge significantly affects decisions, it may be best to assign evaluators with various backgrounds for evaluation.

Similarly, since the previous evaluation experience is critical to decision quality, selecting experienced evaluators and offering them training is also helpful to improve their decision quality. Furthermore, since the increased evaluation efforts of the evaluators improves their ability to identify high-quality proposals, it is crucial to give evaluators a sufficient amount of time to improve the decision quality.

### Limitations and Future Research

These contributions, however, must be qualified in light of two critical limitations of this study. First is the limitation of data. Although the data enabled us to observe how cognitive search behavior was related to decision quality, the data collected in this study did not precisely determine causality in nature. Therefore, we encourage future researchers to use experimental manipulations to explicitly show the causal role of search behavior in influencing decision-making.

Second, the role of different types of organizational structures in decision-making needs to be further explored. This study only selects evaluation data from universities. The characteristics of evaluators from other industries, such as evaluators’ performance from government departments and enterprises, are also worth exploring. Assessing decisions may have unique functions in organizational types, for example, the bureaucratic/orthodox organization, the professional organization, the postmodern organization, the representative democratic organization, and network organizations (Diefenbach and Sillince, 2011).

## Conclusion

Our finding suggests that decision-makers are more confident of their decision with intermediate levels of cognitive proximity. Optimal knowledge proximity is reached when people’s knowledge bases shared similarities and some newness (Nooteboom et al., 2007). Such relation is lessened by evaluation experience as evaluation experience positively affects decision quality, i.e., the curvilinear relationship is less pronounced for evaluators with high evaluation experience than for those with low evaluation experience. Moreover, the inverted U-shaped relationship between cognitive proximity and decision quality is also moderated by evaluation efforts. That is, the curvilinear relationship is less pronounced for evaluators with high evaluation efforts than for those with low evaluation efforts.

## Data Availability Statement

The datasets presented in this article are not readily available because the data belongs to an ongoing project. Requests to access the datasets should be directed to the first author ZC,chuqingzhang@126.com.

## Author Contributions

All authors listed have made a substantial, direct, and intellectual contribution to the work and approved it for publication.

## Funding

This research project is supported by the Science Foundation of Beijing Language and Culture University (supported by the Fundamental Research Funds for the Central Universities) (21YJ050007) and Foshan and Tsinghua Industry-University Research Collaborative Innovation Project (2019THFS01).

## Conflict of Interest

The authors declare that the research was conducted in the absence of any commercial or financial relationships that could be construed as a potential conflict of interest.

## Publisher’s Note

All claims expressed in this article are solely those of the authors and do not necessarily represent those of their affiliated organizations, or those of the publisher, the editors and the reviewers. Any product that may be evaluated in this article, or claim that may be made by its manufacturer, is not guaranteed or endorsed by the publisher.
